# 

**Published:** 1890-07

**Authors:** 


TABLE OF CONTENTS.

ORIGINAL COMMUNICATIONS. PAGE

Oral Deformities and their Correction. By KASSON C. GIBSON, New York, N. Y. . . . . 385

Local Anæsthesia by Nitrous Oxide: A Convenient Method of applying it. By G. L. CURTIS, M.D., D.D.S., Syracuse, N. Y. . . . . 403

REPORTS OF SOCIETY MEETINGS.

Seventh Annual Session of the Maryland State Dental Association . . . . 408

New-York Odontological Society . . . . 420

Odontological Society of Pennsylvania . . . . 433

EDITORIAL.

Special Announcement . . . . 436

Au Revoir . . . . 436

The Conventions . . . . 438

Bibliography . . . . 439

FOREIGN CORRESPONDENCE . . . . 441

DOMESTIC CORRESPONDENCE . . . . 442

CURRENT NEWS . . . .

American Dental Association . . . . 447

Meeting of the American Dental Association . . . . 447

Items . . . . 448

